# Correction: Govaerts et al. Incidence and Clinical Features of Pseudoprogression in Brain Metastases After Immune-Checkpoint Inhibitor Therapy: A Retrospective Study. *Cancers* 2025, *17*, 2425

**DOI:** 10.3390/cancers18040572

**Published:** 2026-02-10

**Authors:** Chris W. Govaerts, Miranda C. A. Kramer, Ingeborg Bosma, Frank A. E. Kruyt, Frederike Bensch, J. Marc C. van Dijk, Mathilde Jalving, Anouk van der Hoorn

**Affiliations:** 1 Department of Medical Oncology, University Medical Center Groningen, University of Groningen, 9713 GZ Groningen, The Netherlands; f.a.e.kruyt@umcg.nl (F.A.E.K.); m.jalving@umcg.nl (M.J.); 2 Department of Radiology, Medical Imaging Center, University Medical Center Groningen, University of Groningen, 9713 GZ Groningen, The Netherlands; a.van.der.hoorn@umcg.nl; 3 Department of Radiation Oncology, University Medical Center Groningen, University of Groningen, 9713 GZ Groningen, The Netherlands; m.c.a.kramer@umcg.nl; 4 Department of Neurology, University Medical Center Groningen, University of Groningen, 9713 GZ Groningen, The Netherlands; i.bosma01@umcg.nl; 5 Department of Pulmonary Diseases and Tuberculosis, University Medical Center Groningen, University of Groningen, 9713 GZ Groningen, The Netherlands; f.bensch@umcg.nl; 6 Department of Neurosurgery, University Medical Center Groningen, University of Groningen, 9713 GZ Groningen, The Netherlands; j.m.c.van.dijk@umcg.nl

## 1. Error in Figure/Table

In the original publication [1], there were mistakes in Table 2 and Figure 5B as published. This is due to several errors in lesion measurement values and calculation due to mistakes made in data handling and the posting of incorrect values, which are updated in the Supplementary Materials (see below). The corrected Table 2 and Figure 5B appear below. 

Changes:

Table 2:

- Under the heading PsP, the longest sagittal diameter at the progression scan has been changed from a median of 14 to 15.
- The Mann–Whitney U value for the longest sagittal diameter at progression scan (TP vs. PsP) *p* has been changed from 0.12 to 0.13.
- The percentage values under the ‘TP’ and ‘PsP’ headings for the ‘pattern of progression prior to progression scan’ variable as given are incorrect and have been rewritten from 24.9 to 14.6 and 23.2 to 13.6.
- Pre-existent changed into Pre-existing.
- 0.19 ^1^ should be in the same row with Emergence of new lesions, n (%).

**Table 2:** Further clinical comparisons at the progression and definitive diagnosis scans. Overview of additional clinical parameters at the points of initial progression and the definitive diagnosis. Lesions are presented according to definitive diagnosis as TP, NC, and PsP. Abbreviations—TP: tumour progression; NC: non-classified; PsP: pseudoprogression; IQR: interquartile range. ^1^: Analyses performed by summing the total cases where new lesions emerged at the progression/definitive diagnosis scans, prior to these scans and both at and prior to these. This total was then compared to the number of cases where no lesions emerged throughout the indicated follow-up points.

	TP (N = 41)	NC (N = 170)	PsP (N = 22)	Mann–Whitney U/Fisher’s Exact *p*-Value TP-PsP
Lesion type, n (%)				0.29
De novo	17 (41.5)	80 (47.1)	6 (27.3)	
Pre-existing	24 (58.5)	90 (52.9)	16 (72.7)	
Emergence of new lesions, n (%)				0.19 ^1^
At progression scan	22 (53.7)	109 (64.1)	8 (36.4)	
Prior to progression scan	1 (2.4)	11 (6.5)	2 (9.1)	
Both prior to and at progression scan	3 (7.3)	6 (3.5)	0 (0.0)	
Emergence of new lesions, n (%)				0.53 ^1^
At definitive diagnosis scan	7 (17.1)	12 (7.1)	4 (18.2)	
Prior to definitive diagnosis scan	0 (0.0)	21 (12.4)	1 (4.5)	
Both prior to and at definitive diagnosis scan	1 (2.4)	2 (1.2)	1 (4.5)	
Pattern of progression prior to progression scan and starting ICI therapy, n (%)				
Yes	6 (14.6)	14 (8.2)	3 (13.6)	>0.99 (<1.00)
Longest transverse diameter (mm), median (IQR)				
Baseline	7 (5–14)	5 (3–8)	7 (6–11)	0.80
Nadir	6 (5–13)	5 (3–7)	7 (4–11)	0.88
Progression	19 (12–32)	13 (11–18)	15 (11–18)	0.06
Definitive diagnosis	23 (19–30)	10 (5–16)	9 (4–11)	<0.01
Longest sagittal diameter (mm), median (IQR)				
Baseline	8 (5–16)	5 (3–8)	8 (5–10)	0.70
Nadir	6 (4–14)	5 (3–7)	7 (4–10)	0.82
Progression	18 (12–31)	14 (11–19)	15 (11–19)	0.13
Definitive diagnosis	23 (19–29)	10 (5–16)	9 (4–11)	<0.01
Longest sum transverse diameter target lesions (mm), median (IQR)				
Baseline	18 (8–34)	19 (6–31)	26 (7–50)	0.82
Nadir	16 (8–31)	15 (5–30)	24 (7–39)	0.93
Progression	31 (19–48)	38 (17–75)	32 (17–60)	0.79
Definitive diagnosis	34 (22–66)	24 (10–40)	12 (7–21)	<0.01
Longest sum sagittal diameter target lesions (mm), median (IQR)				
Baseline	19 (8–31)	19 (6–34)	29 (7–49)	0.69
Nadir	16 (8–30)	15 (6–34)	25 (7–42)	0.71
Progression	31 (18–54)	37 (16–75)	33 (16–55)	0.82
Definitive diagnosis	35 (22–68)	23 (11–41)	12 (7–23)	<0.01

Figure 5B:

- Under ‘definitive diagnosis scan: target lesions’, TP has been changed from n = 43 to 42, and SD has been changed from n = 79 to 80.

**Figure 5:**
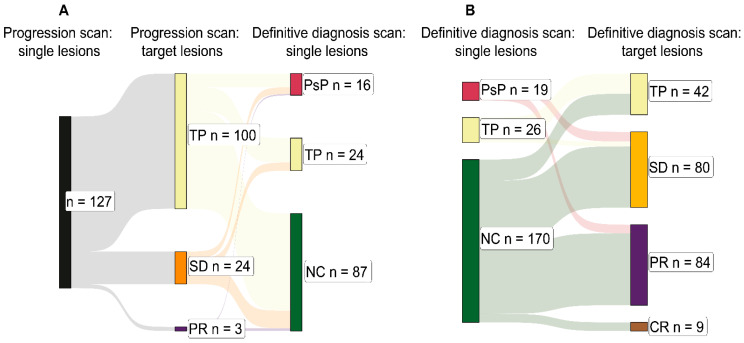
Total intracranial burden at initial progression and definitive diagnosis. (**A**) Sankey chart depicting the relationships between individual lesions under analysis at the point of initial progression—the progression scan (left: black)—and the suggested diagnosis according to the sum longest lesion diameter changes in all target lesions at the progression scan (middle: yellow, orange, and purple). This is followed by the definitive diagnosis for the individual lesions at the definitive diagnosis scan (right: crimson, yellow, and green). Target lesions were summed in the case of each of the 233 lesions included in the study except de novo lesions (no nadir scan available) and lesions diagnosed by histopathology (no definitive diagnosis scan available). This explains the smaller sample size of 127. (**B**) Sankey chart depicting the relationships between individual lesions under analysis at the definitive diagnosis scan (left: crimson, yellow, and green) and the suggested definitive diagnosis according to the sum longest lesion diameter changes in all target lesions at the definitive diagnosis scan (right: yellow, orange, purple, and brown). Similarly, target lesions were summed in the case of each of the 233 lesions included in the study except lesions diagnosed by histopathology (no definitive diagnosis scan available), explaining the smaller sample sizes for PsP (n = 19) and TP (n = 26). Abbreviations—TP: tumour progression; SD: stable disease; PR: partial response; PsP: pseudoprogression; NC: non-classified; CR: complete response.

## 2. Error in Supplementary Materials

In the original publication, there was a mistake in Supplementary Data S1 as published, whereby some data were inadvertently included. In the corrected file, specific treatment start dates and pseudonymized patient codes in three columns have been removed to negate any privacy risk to the participants. In addition, to reduce any residual risk, the number of decimal places for the presented ages has been reduced to 0, and a column providing the age at death has been removed.

There were also several errors in the lesion measurement values due to mistakes made in data handling and the posting of incorrect values. The corrected data points are given below, which were verified by referring to the original lesion measurements made on MRI at the time of analysis. 

Row 2: absol_change_prog_ddx_scan sag has been changed from 20 to 18, with a corresponding percentage change.

Row 21: absol_change_prog_ddx_scan trans and sag have been changed from 4 and 5 to –2 and 6, with corresponding percentage changes.

Row 56: prog_dim_trans and prog_dim_sag have been changed from 14 × 12 and 14 × 11 to 18 × 13 and 16 × 13.

Row 120: absol_change_prog_ddx_scan sag has been changed from −14 to −13 with a corresponding percentage change.

Row 138: absol_change_prog_ddx_scan trans has been changed from 16 to 14 with a corresponding percentage change.

Row 139: prog_dim trans and prog_dim sag has been changed from 8 × 7 and 10 × 6 to 11 × 10 and 10 × 8.

Row 148: ddx_dim sag has been changed from 25 × 15 to 26 × 15; ddx_dim_target sag has been changed from 25 × 15 to 26 × 15.

Row 227: ddx_dim trans and sag have been changed from 25 × 20 and 24 × 23 to 20 × 14 and 18 × 12; ddx_dim_target trans and sag have been changed from 24 × 20 and 24 × 23 to 20 × 14 and 18 × 12.

## 3. Error in Abstract

Due to a typing error in the abstract, the line in the results section ‘A cohort of 98 patients with 233 lesions was included over a 13-year period’ should read ‘A cohort of 98 patients with 233 lesions was included over an 11-year period’.

The authors state that the scientific conclusions are unaffected. This correction was approved by the Academic Editor. The original publication has also been updated.

## References

[1] Govaerts C.W., Kramer M.C.A., Bosma I., Kruyt F.A.E., Bensch F., van Dijk J.M.C., Jalving M., van der Hoorn A. (2025). Incidence and Clinical Features of Pseudoprogression in Brain Metastases After Immune-Checkpoint Inhibitor Therapy: A Retrospective Study. Cancers.

