# Supplementary material for: Incidence and Clinical Features of Pseudoprogression in Brain Metastases After Immune-Checkpoint Inhibitor Therapy: A Retrospective Study
Source: Cancers (Basel). 2025 Jul 22;17(15):2425. doi: 10.3390/cancers17152425 (PMC12346240; doi:10.3390/cancers17152425)
Supplement: Supplementary file 1 [file cancers-17-02425-s001.zip › Supplementary_Figures.pdf]

Incidence and Clinical Features of Pseudoprogression in Brain Metastases After Immune-Checkpoint Inhibitor Therapy: A Retrospective Study

Chris W. Govaerts [1,2,*], Miranda C. A. Kramer [3], Ingeborg Bosma [4], Frank A. E. Kruyt [1], Frederike Bensch [5], J. Marc C. van Dijk [6], Mathilde Jalving [1] and Anouk van der Hoorn [2]

[1] Department of Medical Oncology, University Medical Center Groningen, University of Groningen, 9713 GZ Groningen, The Netherlands; f.a.e.kruyt@umcg.nl (F.A.E.K.); m.jalving@umcg.nl (M.J.)
[2] Department of Radiology, Medical Imaging Center, University Medical Center Groningen, University of Groningen, 9713 GZ Groningen, The Netherlands; a.van.der.hoorn@umcg.nl
[3] Department of Radiation Oncology, University Medical Center Groningen, University of Groningen, 9713 GZ Groningen, The Netherlands; m.c.a.kramer@umcg.nl
[4] Department of Neurology, University Medical Center Groningen, University of Groningen, 9713 GZ Groningen, The Netherlands; i.bosma01@umcg.nl
[5] Department of Pulmonary Diseases and Tuberculosis, University Medical Center Groningen, University of Groningen, 9713 GZ Groningen, The Netherlands; f.bensch@umcg.nl
[6] Department of Neurosurgery, University Medical Center Groningen, University of Groningen, 9713 GZ Groningen, The Netherlands; j.m.c.van.dijk@umcg.nl
* Correspondence: c.w.govaerts@umcg.nl

SUPPLEMENTARY FIGURES

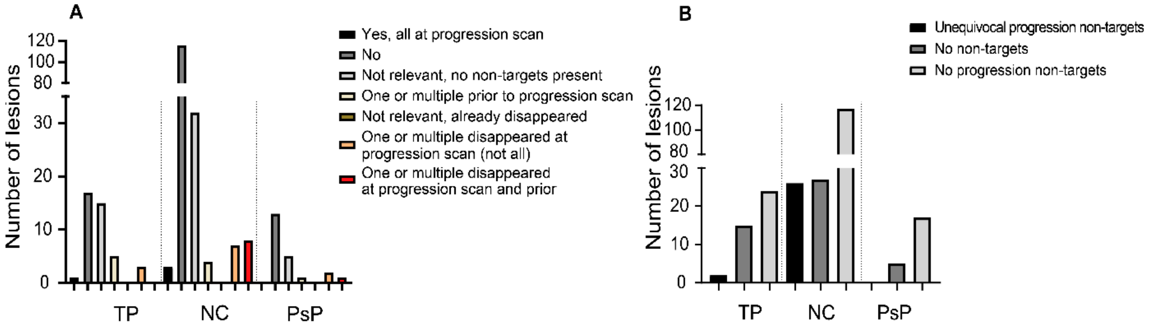

Supplemental Figure S1. Behaviour of non-target lesions at the progression scan.
(A) Bar plot indicating whether there was disappearance of non-target lesions at the progression scan. The bars are coloured according to whether in the context of the lesion in question, all non-targets disappeared (black), no non-targets disappeared (grey), there were no non-targets present throughout follow-up (light grey), one or multiple disappeared but prior to the progression scan and after the baseline scan (beige), all non-target lesions disappeared prior to the progression scan but after the baseline scan (brown), one or multiple (but not all) disappeared at the progression scan itself (orange) and one or multiple (but not all) disappeared at both the progression scan and prior to it (red). (B) Bar plot showing whether there was unequivocal progression of all non-target lesions present (black) according to RANO-BM criteria at the progression scan. Not-relevant (grey) is where no non-targets were present.
Abbreviations- TP: tumour progression; NC: non-classified; PsP: pseudoprogression

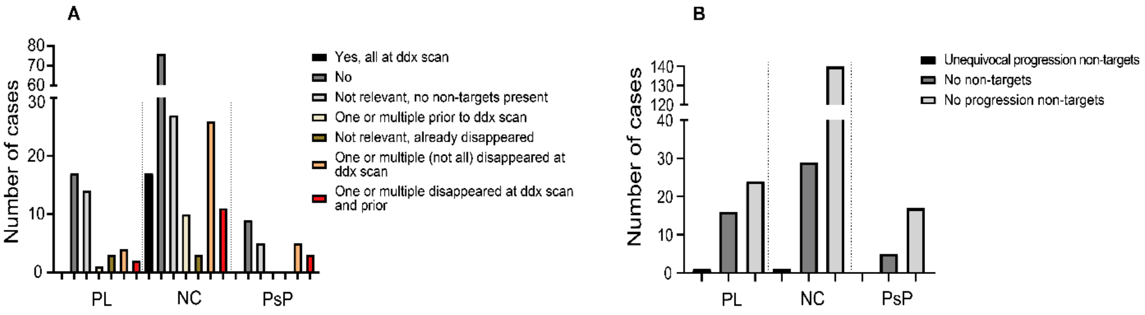

Supplemental Figure S2. Behaviour of non-target lesions at the definitive diagnosis scan. (A) Bar plot indicating whether there was disappearance of non-target lesions at the definitive diagnosis scan. The bars are coloured according to whether in the context of the lesion in question, all non-targets disappeared (black), no non-targets disappeared (dark grey), there were no non-targets present throughout follow-up (light grey), one or multiple disappeared but prior to the definitive scan and after the progression scan (beige), all non-target lesions disappeared at any point prior to the definitive diagnosis scan (brown), one or multiple (but not all) disappeared at the definitive diagnosis scan itself (orange) and one or multiple (but not all) disappeared at both the definitive diagnosis scan and at any point prior to it (red). (B) Bar plot showing whether there was unequivocal progression of all non-target lesions present (black) according to RANO-BM criteria at the definitive scan. Not-relevant (grey) is where no non-targets were present.
Abbreviations- TP: tumour progression; NC: non-classified; PsP: pseudoprogression

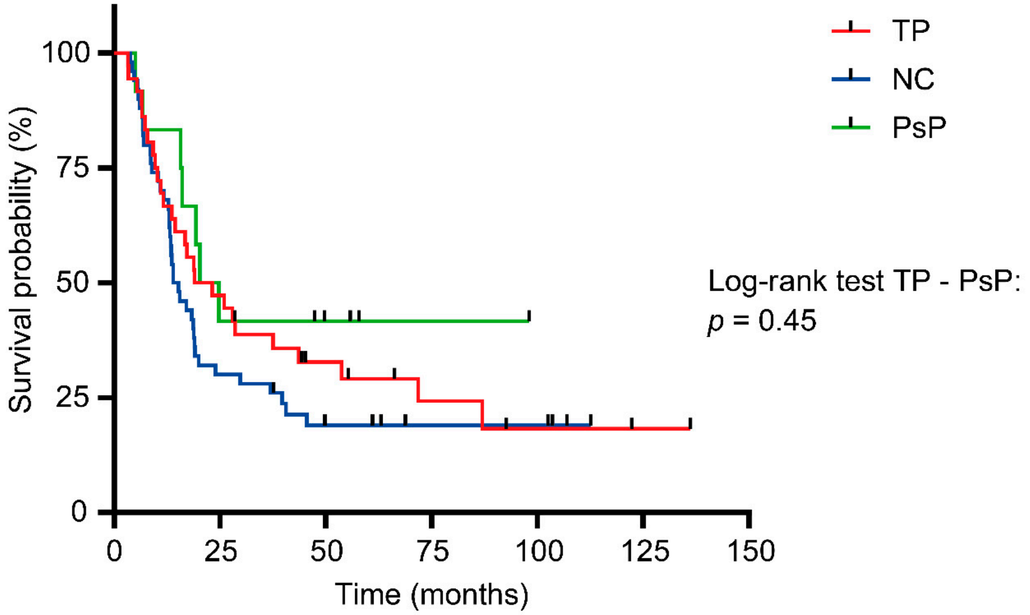

Number at risk

	0	25	50	75	100	125	150
TP	36	18	10	5	3	2	1
NC	50	16	8	5	5	1	1
PsP	12	6	4	2	1	1	1

Time (months)

Supplemental Figure S3. Kaplan-Meier curves by diagnostic group

Kaplan-Meier curves of overall survival per patient from the point of the progression scan in months according to the TP, NC and PsP diagnostic groups. Censored patients are indicated with vertical black lines on the curves. The p-value for the Log-rank test between TP and PsP is indicated

Abbreviations- TP: tumour progression; NC: non-classified; PsP: pseudoprogression.
