# Supplementary material for: Incidence and Clinical Features of Pseudoprogression in Brain Metastases After Immune-Checkpoint Inhibitor Therapy: A Retrospective Study
Source: Cancers (Basel). 2025 Jul 22;17(15):2425. doi: 10.3390/cancers17152425 (PMC12346240; doi:10.3390/cancers17152425)
Supplement: Supplementary file 1 [file cancers-17-02425-s001.zip › Supplementary_Table_S1.pdf]

Incidence and Clinical Features of Pseudoprogression in Brain Metastases After Immune-Checkpoint Inhibitor Therapy: A Retrospective Study

Chris W. Govaerts [1,2,*], Miranda C. A. Kramer [3], Ingeborg Bosma [4], Frank A. E. Kruyt [1], Frederike Bensch [5], J. Marc C. van Dijk [6], Mathilde Jalving [1] and Anouk van der Hoorn [2]

[1] Department of Medical Oncology, University Medical Center Groningen, University of Groningen, 9713 GZ Groningen, The Netherlands; f.a.e.kruyt@umcg.nl (F.A.E.K.); m.jalving@umcg.nl (M.J.)
[2] Department of Radiology, Medical Imaging Center, University Medical Center Groningen, University of Groningen, 9713 GZ Groningen, The Netherlands; a.van.der.hoorn@umcg.nl
[3] Department of Radiation Oncology, University Medical Center Groningen, University of Groningen, 9713 GZ Groningen, The Netherlands; m.c.a.kramer@umcg.nl
[4] Department of Neurology, University Medical Center Groningen, University of Groningen, 9713 GZ Groningen, The Netherlands; i.bosma01@umcg.nl
[5] Department of Pulmonary Diseases and Tuberculosis, University Medical Center Groningen, University of Groningen, 9713 GZ Groningen, The Netherlands; f.bensch@umcg.nl
[6] Department of Neurosurgery, University Medical Center Groningen, University of Groningen, 9713 GZ Groningen, The Netherlands; j.m.c.van.dijk@umcg.nl
***** Correspondence: c.w.govaerts@umcg.nl

SUPPLEMENTARY TABLE

Supplemental Table S1. Molecular diagnostic and systemic treatment information
Additional molecular diagnostic information and details on systemic treatment prior to the progression scan. Lesions are presented according to definitive diagnosis as TP, NC and PsP. Under ‘prior systemic or targeted therapy’, the number denomination prior to the therapy abbreviation (e.g. 1-MTT: *see below*) refers to the number of variants of the administered treatment.
Abbreviations- TP: tumour progression; NC: non-classified; PsP: pseudoprogression; DCT: dual-chemotherapy (e.g. carboplatin + pemetrexed); MCT: mono-chemotherapy (e.g. dacarbazine); TCT: triple-chemotherapy (e.g. doxorubicin + cyclophosphamide + paclitaxel); MTT: mono-targeted-therapy (e.g. vemurafenib, lapatinib, alectinib); DTT: dual-targeted-therapy (e.g. dabrafenib + trametinib); HT: hormonal therapy (e.g. tamoxifen); VEGF: anti-vascular endothelial growth factor treatment (e.g. bevacizumab); MAT: other monoclonal antibody treatment (e.g. canakinumab)
†: administered for previously diagnosed breast cancer

	**TP (N = 41)**	**NC (N = 170)**	**PsP (N = 22)**	**Mann-Whitney U p-value TP - PsP**
Mutation status primary tumour/extracranial metastatic site, n (%)				
BRAF p.K601E	0 (0.0)	2 (1.2)	1 (4.5)	
BRAF p.T599	1 (2.4)	5 (2.9)	0	
BRAF p.V600E	21 (51.2)	104 (61.2)	11 (50.0)	
BRAF p.V600K	1 (2.4)	13 (7.6)	0	
IDH1 p.R132C	0 (0.0)	1 (0.6)	0	
EGFR p.L858R	1 (2.4)	0 (0.0)	0	
KIT p.K624E	1 (2.4)	0 (0.0)	1 (4.5)	
KIT p.W557R	0 (0.0)	0 (0.0)	1 (4.5)	
KRAS p.G12A	0 (0.0)	1 (0.6)	0	-
KRAS p.G12C	1 (2.4)	8 (4.7)	0	
KRAS p.G12D	0 (0.0)	4 (2.4)	1 (4.5)	
KRAS p.G12V	0 (0.0)	1 (0.6)	0	
NRAS p.Q61H	1 (2.4)	2 (1.2)	0	
NRAS p.Q61K	2 (4.9)	4 (2.4)	1 (4.5)	
NRAS p.Q61R	2 (4.9)	10 (5.9)	0	
PIK3CA p.E545K	1 (2.4)	1 (0.6)	0	
PIK3CA p.E542K	0 (0.0)	1 (0.6)	0	
PIK3CA p.H1047R	0 (0.0)	0 (0.0)	1 (4.5)	
No mutation found	9 (22.0)	13 (7.6)	5 (22.7)	

	24 (58.5)	127 (74.7)	14 (63.6)	
Prior systemic or targeted therapy, n (%)	3 (7.3)	7 (4.1)	2 (9.1)	
1-DCT	0 (0.0)	2 (1.2)	0 (0.0)	
2-DCT	1 (2.4)	1 (0.6)	1 (4.5)	
1-MCT	3 (7.3)	20 (11.8)	5 (22.7)	
1-MTT	8 (19.5)	66 (38.8)	4 (18.2)	
1-DTT	1 (2.4)	4 (2.4)	0 (0.0)	
2-DTT	0 (0.0)	2 (1.2)	0 (0.0)	
3-DTT	2 (4.9)	4 (2.4)	0 (0.0)	
1-DCT & 1-MCT	2 (4.9)	3 (1.8)	1 (4.5)	-
1-MCT & 1-MTT	0 (0.0)	1 (0.6)	0 (0.0)	
1-MCT & 1-DTT	2 (4.9)	9 (5.3)	0 (0.0)	
1-MTT & 1-DTT	0 (0.0)	2 (1.2)	1 (4.5)	
1-TCT & 1-HT[†]	0 (0.0)	2 (1.2)	0 (0.0)	
1-DCT & 1-MCT & 1-DTT				
2-MTT & 1-DCT+1-VEGF & 1-VEGF	2 (4.9)	1 (0.6)	0 (0.0)	
1-DCT+1-MAT & 1-MCT+1-MAT				
1-DCT+1-VEGF & 1-VEGF & 1-MTT	0 (0.0)	2 (1.2)	0 (0.0)	
	0 (0.0)	1 (0.6)	0 (0.0)	
